# Optimizing Postoperative Outcomes in Abdominal Surgery: The Role of Enhanced Recovery After Surgery (ERAS) Protocols

**DOI:** 10.7759/cureus.79258

**Published:** 2025-02-18

**Authors:** Younis Mohamed, Ahmed Hussein, Omar Elsaba, Mahmoud Rhodes, Khalid Alloush, Eman Elhofy, Ahmed Shokry

**Affiliations:** 1 General Surgery, Betsi Cadwaladr University Health Board, Bangor, GBR; 2 Orthopedic Surgery, Alexandria University Hospitals, Alexandria, EGY; 3 Urology, Imbaba General Hospital, Giza, EGY; 4 Trauma and Orthopaedics, Nasser Institute Hospital for Research and Treatment, Cairo, EGY; 5 Obstetrics and Gynaecology, Ain Shams University Hospital, Cairo, EGY; 6 General Surgery, Nottingham University Hospitals NHS Trust, Nottingham, GBR; 7 Trauma and Orthopedic, Aneurin Bevan University Health Board, Newport, GBR

**Keywords:** acute abdominal surgery, eras protocols, patients satisfaction, predictors of post-operative adverse oucomes, recovery rate

## Abstract

Background: The enhanced recovery after surgery (ERAS) protocols aim to reduce surgical stress, enhance recovery, and minimize the length of hospital stays, thereby improving both clinical outcomes and the overall patient experience. The main objective of the study was to find the postoperative outcomes of abdominal surgery with respect to the role of ERAS protocols.

Methodology: Data was* *collected retrospectively from three governmental hospitals in Egypt between 2018 and 2020. A total of 1473 patients were enrolled according to the criteria of the study, 780 in the ERAS group and 693 in the non-ERAS group.

Results: The mean age of patients in both groups was similar, with 55.4 (±10.2) years for the ERAS group and 54.8 (±9.8) years for the non-ERAS group. The gender distribution showed a slightly higher number of female patients in both groups. The average BMI was comparable between groups, with 26.3±4.5 kg/m² in the ERAS group and 26.5±4.7 kg/m² in the non-ERAS group. The time to first flatus was reduced from 52.3 (±10.4) hours in the non-ERAS group to 36.2±8.1 hours in the ERAS group (P < 0.001). Similarly, the time to first defecation was shorter in the ERAS group at 48.5±9.2 hours compared to 66.4±12.5 hours in the non-ERAS group (P < 0.001).

Conclusion: ERAS protocols significantly improve postoperative outcomes in abdominal surgeries by reducing recovery times, complications, and hospital stays.

## Introduction

The management of patients undergoing abdominal surgery has evolved significantly over the past few decades, shifting from traditional recovery approaches to more evidence-based, patient-centered strategies. Several significant developments have occurred in the mentioned area, and one of them is the invention and application of the enhanced recovery after surgery (ERAS) technique. Such measures include seeking to decrease the amount of concern that surgery may cause, expedite recovery, and shorten durations of hospitalization making both the results from surgical intervention as well as perceptions of the general hospital experience optimal [1].

ERAS was first established in the 1990s but has become well-accepted because of favorable results in postoperative care of multiple specialties, especially in the field of abdominal surgery [2]. ERAS protocols are ‘time-based’ and multidisciplinary, in that they are implemented before, during, and after surgery [3]. The concept of ERAS is realized based on the idea that surgical interventions negatively affect the patient's function, and, therefore, all the changes should be reduced to a minimum and the body has to restore its function as soon as possible. The application of the concept of ERAS in abdominal surgery has been shown to come with diverse benefits [4].

Different investigations have confirmed that patients who receive ERAS-implemented treatments recover faster, have fewer complications, and have fewer hospital days than those who receive conventional care [5]. Such enhancements might be associated with the ERAS protocol since this strategy implies a global optimization of patient management processes. For instance, frameworks such as early mobilization and nutritional support aid in the preservation of muscles and immunity and, hence, the low risk of infections or other complications after surgery [6]. The occurrence of postoperative complications creates severe healthcare burdens for patients and healthcare systems. Previous studies found patients who acquire surgical postoperative complications pay more than patients who do not experience complications [1-4]. Hospital stays of longer duration account for most of these costs since additional resources become necessary [1,7]. Researchers have extensively worked to boost awareness levels and combat the occurrence of postoperative complications. The occurrence of postoperative complications also creates severe healthcare burdens for patients and healthcare systems.

However, ERAS also has some limitations; the most important barrier involves the implementation of a comprehensive plan of care, where surgeons, anesthesiologists, nurses, nutritionists, and physiotherapists among other personnel must work together [8]. It takes time and money for hospitals to effectively train their staff and all members of a healthcare team in ERAS protocols [9]. Another difficulty is patients cannot be relied upon to strictly follow the practiced protocol. Those aspects of ERAS such as early feeding and mobilization are helpful; however, this can be uncomfortable for patients in comparison with traditional postoperative practices [10].

ERAS delivers multimodal surgical care techniques that function as preoperative management approaches to speed recovery times by supporting intact organ operation and diminishing intense postoperative body stress. ERAS protocols combine preoperative counseling along with nutritional optimization, standardize both analgesic administration and anesthesia techniques, and facilitate early patient mobilization [11]. The established ERAS protocol operates successfully in elective surgery while also enabling its use across all GI and non-GI surgical procedures [12].

The main objective of the study is to find the optimizing postoperative outcomes in abdominal surgery and the role of ERAS protocols.

## Materials and methods

Data was collected retrospectively from three government hospitals (Nasser Institute for Research and Treatment, Al Agouza Hospital, and Al Haram Hospital) in Cairo, Egypt, between February 2018 and December 2020.

Inclusion and exclusion criteria

The inclusion criteria were adult patients (≥18 years) undergoing elective abdominal surgery. The surgeries included gynecological, general, colorectal, and urological procedures, with patients ranging from American Society of Anesthesiologists (ASA) scores I-III, ensuring a manageable level of comorbidities. The exclusion criteria involved patients under 18 years of age, emergency surgeries, severe systemic disease (ASA IV or higher), pregnancy, chronic opioid use, severe malnutrition, and psychiatric or cognitive disorders. 

A total of 1473 patients were included according to the above criteria of which 780 patients had been managed according to the ERAS protocols and 693 patients without ERAS management were included as the non-ERAS group.

Data collection and outcomes

Patient data were collected retrospectively with a focus on postoperative outcomes, adherence to ERAS protocols, and follow-up over defined periods. Data were collected from different sources, including medical records, operative notes, quality department records, anesthetic notes, preoperative assessment notes, postoperative recovery unit notes, archives, and operating room records. 

The primary outcomes included the following metrics, recorded during the 30-day, 90-day, 120-day, and six-month follow-ups: overall health status, chronic issues, and quality of life, particularly in oncological procedures. ERAS protocols include several preoperative, intraoperative, and postoperative components aimed at enhancing recovery. The outcomes were measured to assess the protocol's effectiveness. Time to first flatus as an indicator of bowel function recovery and time to first defecation as another key indicator of the return of gastrointestinal function were noted. Time to first oral liquid and solid diet and time to ambulation after surgery was also measured by the promptness of patient mobility, a key component of the ERAS protocol. Length of hospital stay (LOS) includes the total duration of the patient's hospital stay after surgery. ERAS protocols were also assessed in relation to the moderation of postoperative outcomes such as major complications, pulmonary complications, anastomosis leakage (in patients who underwent anastomosis), paralytic ileus, surgical site infection, and medical logic module (MLM). Although the services offered were free of charge, hence failing to include hospital costs, the study relied on clinical results and resource use.

Statistical analysis

Data were analyzed using IBM SPSS Statistics for Windows, Version 26.0 (2019; IBM Corp., Armonk, New York, United States). The study analyzed the effectiveness of ERAS protocols by comparing patients' outcomes, including hospital stay duration, return of physiological functions, and complication rates. Readmission rates were also evaluated. Chi-square test was applied to compare the outcomes between both groups. A p-value of <0.05 were considered as significant.

## Results

A total of 1473 patients were included in this study with 780 patients in the ERAS group and 693 in the non-ERAS group. The mean age of patients in both groups was similar, with 55.4 (±10.2) years for the ERAS group and 54.8 (±9.8) years for the non-ERAS group. The gender distribution showed a slightly higher number of female participants in both groups. The average BMI was comparable between groups, with 26.3 (±4.5) kg/m² in the ERAS group and 26.5 (±4.7) kg/m² in the non-ERAS group. ASA scores and comorbidity rates were also similar, with 42% of ERAS patients and 45% of non-ERAS patients reporting comorbid conditions (Table 1).

**Table 1:** Demographic data of participants (N=1473) ERAS: Enhanced Recovery After Surgery

Characteristic	ERAS Group (n=780)	Non-ERAS Group (n=693)
Age (years), mean ± SD	55.4 ± 10.2	54.8 ± 9.8
Sex, n (%)		
Male	346 (44.4%)	324 (46.8%)
Female	434 (55.6%)	369 (53.2%)
BMI (kg/m²), mean ± SD	26.3 ± 4.5	26.5 ± 4.7
ASA Score, n (%)		
I	320 (41%)	310 (44.8%)
II	300 (38.5%)	280 (40.4%)
III	160 (20.5%)	103 (14.9%)
Comorbidities, n (%)	328 (42%)	312 (45%)
Hypertension	218 (28%)	208 (30%)
Diabetes Mellitus	109 (14%)	111 (16%)
Cardiovascular Disease	78 (10%)	83 (12%)
Chronic Kidney Disease	39 (5%)	42 (6%)
Pulmonary Disease	62 (8%)	62 (9%)
Oncological Procedures, n (%)	273 (35%)	263 (38%)

The time to first flatus was reduced from 52.3 (±10.4) hours in the non-ERAS group to 36.2 (±8.1) hours in the ERAS group (P < 0.001). Similarly, the time to first defecation was shorter in the ERAS group at 48.5 (±9.2) hours compared to 66.4 (±12.5) hours in the non-ERAS group (P < 0.001). Additionally, the ERAS group had a faster return to oral intake, with time to first oral liquid diet and solid diet significantly reduced compared to the non-ERAS group (P < 0.001 for both) (Table 2).

**Table 2:** Primary outcomes and complications (N=1473) ERAS: Enhanced Recovery After Surgery

Outcome/Complication	ERAS Group (n=780), mean± SD	Non-ERAS Group (n=693), mean± SD	P-value	Degrees of Freedom (df)	Effect Size (Cramér's V)
Time to First Flatus (hours)	36.2 ± 8.1	52.3 ± 10.4	< 0.001	1	0.17
Time to First Defecation (hours)	48.5 ± 9.2	66.4 ± 12.5	< 0.001	1	0.18
Time to First Oral Liquid Diet (hours)	12.8 ± 3.4	24.6 ± 5.1	< 0.001	1	0.20
Time to First Oral Solid Diet (hours)	48.2 ± 8.6	72.5 ± 11.8	< 0.001	1	0.19

Time to first urination was reduced to 15.3 (± 5.2) hours in the ERAS group, compared to 27.4 (± 7.6) hours in the non-ERAS group (P < 0.001). Similarly, the time to first ambulation was faster in the ERAS group at 14.5 (± 4.5) hours versus 28.7 (± 6.9) hours in the non-ERAS group (P < 0.001). The ERAS group also experienced quicker urinary catheter removal at 24.6 (± 6.1) hours compared to 38.3 (± 8.4) hours in the non-ERAS group (P < 0.001), and tolerated oral solid food earlier at 48.2 (± 8.6) hours versus 72.5 (± 11.8) hours in the non-ERAS group (P < 0.001) (Table 3).

**Table 3:** Time to return of function (N=1473) ERAS: Enhanced Recovery After Surgery

Functional Milestone	ERAS Group (n=780), mean± SD	Non-ERAS Group (n=693), mean± SD	P-value	Degrees of Freedom (df)	Effect Size (Cramér's V)
Time to First Urination	15.3 ± 5.2	27.4 ± 7.6	< 0.001	1	0.19
Time to First Ambulation	14.5 ± 4.5	28.7 ± 6.9	< 0.001	1	0.18
Time to Removal of Urinary Catheter	24.6 ± 6.1	38.3 ± 8.4	< 0.001	1	0.17
Time to Tolerating Oral Solid Diet	48.2 ± 8.6	72.5 ± 11.8	< 0.001	1	0.20

Minor complications (Grade I) occurred in 4.3% of ERAS patients versus 7.1% in the non-ERAS group (P = 0.009). Similarly, moderate complications (Grade II) were less frequent in the ERAS group at 3.2% compared to 5.8% in the non-ERAS group (P = 0.017). Surgical intervention-required complications (Grade III) were also lower in the ERAS group at 5.6% versus 8.1% in the non-ERAS group (P = 0.015). Life-threatening complications (Grade IV) were reduced in the ERAS group (1.2%) compared to the non-ERAS group (2.5%) (P = 0.048), while mortality rates (Grade V) were similar between both groups (P = 0.68) (Table 4).

**Table 4:** Severity of postoperative complications (Clavien-Dindo Classification) (N=1473) ERAS: Enhanced Recovery After Surgery

Complication Severity (Clavien-Dindo)	ERAS Group (n=780), n (%)	Non-ERAS Group (n=693), n (%)	P-value	Degrees of Freedom (df)	Effect Size (Cramér's V)
Grade I (Minor complications)	33 (4.3%)	49 (7.1%)	0.009	1	0.06
Grade II (Moderate complications)	25 (3.2%)	40 (5.8%)	0.017	1	0.05
Grade III (Requires surgical intervention)	44 (5.6%)	56 (8.1%)	0.015	1	0.05
Grade IV (Life-threatening)	9 (1.2%)	17 (2.5%)	0.048	1	0.04
Grade V (Death)	12 (1.5%)	12 (1.7%)	0.68	1	0.01

At 30 days postoperatively, the ERAS group had a mean quality of life score of 75.6± 10.3, compared to 68.4±12.7 in the non-ERAS group (P = 0.002) (Table 5). At 90 days, the ERAS group continued to show improved outcomes with a score of 82.2±9.5 versus 72.9±11.3 for the non-ERAS group (P < 0.001). By six months, the quality of life scores further improved to 88.7±8.6 in the ERAS group compared to 79.3±10.5 in the non-ERAS group (P < 0.001). While 12.4% (97 patients) in the ERAS group experienced readmission, only 8.3% (58 patients) in the non-ERAS group were readmitted (Table 6).

**Table 5:** Patient-reported outcomes (quality of life) (N=1473) ERAS: Enhanced Recovery After Surgery

Time Point	ERAS Group (n=780), mean± SD	Non-ERAS Group (n=693), mean± SD	P-value	Degrees of Freedom (df)	Effect Size (Cramér's V)
30 Days Postoperatively	75.6 ± 10.3	68.4 ± 12.7	0.002	1	0.10
90 Days Postoperatively	82.2 ± 9.5	72.9 ± 11.3	< 0.001	1	0.12
180 Days Postoperatively	88.7 ± 8.6	79.3 ± 10.5	< 0.001	1	0.13

**Table 6:** Readmission rate in both groups (N=1473) ERAS: Enhanced Recovery After Surgery

Characteristic	ERAS Group (n=780), n (%)	Non-ERAS Group (n=693), n (%)
Postoperative Readmission within 30 Days	97 (12.4%)	58 (8.3%)

## Discussion

The implementation of ERAS protocols in abdominal surgery has demonstrated significant improvements in postoperative outcomes compared to traditional recovery methods. This cross-sectional study conducted at three governmental hospitals with 1,473 patients has offered useful information on the feasibility of ERAS protocols in minimizing the time to return to activities of daily living, postoperative complications, and hospital stay. 

Many studies show that the recovery rate back to the baseline physiological state is much better in the ERAS group than in the non-ERAS group [13,14]. The patients in the current study whose care followed ERAS plans had shorter times to first flatus, first bowel movement, and intake of oral nutrition; in other words, quicker improvement in gastrointestinal function. This can be explained by the elements of the ERAS program such as early mobilization, limited opioid consumption, and stress on early initiation of oral intake aimed at providing faster restoration of gut function. These results are in accordance with previous literature, which mentions improved functional activity rates as one of the main advantages of ERAS programs [14]. 

The findings of our study indicate that ERAS protocols were linked with reduced overall complication rates, including pulmonary complications, paralytic ileus, and surgical site infections, similar to the study by Giannarini et al. [15]. A number of these potential factors are incorporated into the ERAS pathway such as multimodal pain management that reduces the use of opioids in patients, assessment and optimization of patients' fluid status, and early postoperative mobilization. These effective and efficient strategies minimize postoperative lung compromise, promote normal gastrointestinal activity, and decrease the rate of postoperative surgical site infections. These outcomes are in concordance with earlier studies which have shown that ERAS reduces morbidity after surgery located in the abdomen [16]. 

Another result of the current study that can be discussed is the fact that patients under ERAS management stayed in the hospital for 4.8 days on average while patients in the non-ERAS group averaged 7.6 days. When patients are discharged early, the utilization of assets and resources such as hospital beds and staff is minimized [17]. One of the difficulties noted in the implementation of ERAS protocols in this study involved a higher number of patients who were readmitted within one month of the surgery after discharge as compared to patients who were managed under conventional recovery processes. The slightly higher readmission rate observed in the ERAS group (12.4% vs 8.3%) may be due to early discharge on time given by ERAS, which while reducing the LOS can lead to patients getting readmitted to the hospital for slight complications that might have been managed with more time in the initial hospital stay [18]. Nonetheless, these statistics raise the issue of whether the increased readmission rates are associated with higher readmission rates based on reoperation and mortality and it is seen that these readmissions are most likely due to minor complications that were not fatal or stemmed from complications of surgery [19]. The mortality and re-operation rates were not significantly different between the ERAS and non-ERAS groups suggesting that while implementing ERAS protocols will only enhance patients’ recovery process and reduce complications, it does not increase the risk of mortility or re-operation. This suggests that ERAS protocols are safe, and further calls for widespread implementation, especially among patients with planned elective abdominal surgery [20].

However, some potential limitations affect this study. First, the data collected were retrospective, and this causes selection bias. Also, with the majority of the hospitals belonging to the government, the direct cost of hospital stay cannot be accurately determined, putting drawbacks on the complete economic analysis of the usefulness of implementing ERAS protocols. The next concern is an increased readmission rate in the ERAS group, which should have been investigated in greater detail but it was not the major focus of the study; more research is needed to explore factors that may increase the readmission rate.

## Conclusions

ERAS protocols significantly improve postoperative outcomes in abdominal surgeries by reducing recovery times, complications, and hospital stays. However, the increased readmission rates highlight the need for enhanced postoperative monitoring and support or change of method or approach or patient education. Overall, ERAS protocols provide substantial benefits, making this a valuable approach to optimizing patient recovery and resource utilization.
