# Correction: Optimizing Postoperative Outcomes in Abdominal Surgery: The Role of Enhanced Recovery After Surgery (ERAS) Protocols

**DOI:** 10.7759/cureus.c354

**Published:** 2025-10-09

**Authors:** Younis Mohamed, Ahmed Hussein, Omar Elsaba, Mahmoud Rhodes, Khalid Alloush, Eman Elhofy, Ahmed Shokry

**Affiliations:** 1 General Surgery, Betsi Cadwaladr University Health Board, Bangor, GBR; 2 Orthopedic Surgery, Alexandria University Hospitals, Alexandria, EGY; 3 Urology, Imbaba General Hospital, Giza, EGY; 4 Trauma and Orthopaedics, Nasser Institute Hospital for Research and Treatment, Cairo, EGY; 5 Obstetrics and Gynaecology, Ain Shams University Hospital, Cairo, EGY; 6 General Surgery, Nottingham University Hospitals NHS Trust, Nottingham, GBR; 7 Trauma and Orthopedic, Aneurin Bevan University Health Board, Newport, GBR

This article has been revised to change the affiliation for Khalid Alloush. His department has been changed from Obstetric and Gynecology, Alexandria University Hospitals, Alexandria, Egypt to Obstetrics and Gynaecology, Ain Shams University Hospital, Cairo, Egypt.

